# Quality of life in community-dwelling Dutch elderly measured by EQ-5D-3L

**DOI:** 10.1186/s12955-016-0577-5

**Published:** 2017-01-06

**Authors:** Marie-Josée J. Mangen, Marieke Bolkenbaas, Susanne M. Huijts, Cornelis H. van Werkhoven, Marc J. M. Bonten, G. Ardine de Wit

**Affiliations:** 1 Julius Center for Health Sciences and Primary Care, University Medical Center Utrecht, Str 6.131 room 7.115, Heidelberglaan 100, Utrecht, 3584 CX The Netherlands; 2 Department of Respiratory Medicine, University Medical Center Utrecht, Utrecht, The Netherlands; 3 Department of Medical Microbiology, University Medical Center Utrecht, Utrecht, The Netherlands

**Keywords:** Health-related quality-of-life, Health status, EQ-5D-3L, Elderly, Community-dwelling, The Netherlands

## Abstract

**Background:**

We aimed to evaluate health status and associated factors in community-dwelling elderly in the Netherlands.

**Methods:**

Participants from a placebo-controlled double-blind randomized controlled trial conducted in the Netherlands were invited at the time of enrolment to participate in this study. Data were collected on comorbidities, socio-demographic background and health status, using EQ-5D-3L instrument. EQ-5D-3L summary index values (EQ-5D-indices) was derived using Dutch tariff. Regression analysis was conducted to identify factors associated with EQ-5D-indices and visual analogue scale (EQ-VAS).

**Results:**

48,634 elderly (≥65 years) were included. The most frequently reported complaint was pain/discomfort (29.4%), but for the elder elderly (i.e. ≥85 years) it was mobility (52.9%). The proportion of persons reporting (multiple) problems increased with age from 31.5% for 65–69 years old subjects to 65.9% for elder elderly. The mean EQ-5D-indices and EQ-VAS decreased with age from 0.94 and 84, respectively in those 65 to 69 years old to 0.86 and 76, respectively, in ≥85 years old subjects. Increasing age, female gender, low education, geographic factors and comorbidities were associated with impaired health status.

**Conclusions:**

Within community-dwelling elderly large differences in health status exist. Impairment increases rapidly with age, but health status is also associated with socio-demographic variables and comorbidities.

**Trial registration:**

ClinicalTrials.gov, NCT00812084.

**Electronic supplementary material:**

The online version of this article (doi:10.1186/s12955-016-0577-5) contains supplementary material, which is available to authorized users.

## Background

In 2050 more than 25% of the Dutch population will be aged 65 years and older [1]. With an ageing population and rising health expenditure, preventing illness in elderly becomes more important. Measuring the health of the population is therefore important to guide health policy decisions, normally aimed at improving health and reducing socioeconomic differences in health. The EQ-5D-3L is one of most commonly applied generic health-related quality-of-life instrument [2, 3], and is suitable for measuring health status within an elderly population [4, 5]. There are six publications (i.e., [6–11]) reporting quality-of-life data using the EQ-5D instrument for Dutch elderly. Stolk et al. [6] reported EQ-5D-3L-index and EQ-visual analogue scale (VAS) scores for three age-classes (i.e. 65–69, 70–75 and ≥75 years) and both sexes based on 254 respondents. Furthermore, Timmers et al. [7] reported the EQ-5D-3L index values for elderly in two age-classes of 50–69 and 70–97 years based on 1311 respondents and using data from the second Dutch National Survey of General Practice (DNSGP-2) conducted in 2001 [9]. Further used Hoeymans et al. [9] the DNSGP-2 cohort to present the EQ-domains for elderly (1008 respondents) in three age-classes (i.e. 60–69, 70–79 and ≥80) and both sexes. Szende et al. [8] reported quality-of-life (i.e. EQ-domains, EQ-5D index and EQ-VAS scores) for two age-classes (i.e. 65–74 and ≥75 years) based on 450 respondents, using data from the European Study of the Epidemiology of Mental Disorders (ESEMeD) study collected between 2001 and 2003 [12]. Further used König et al. [10] the ESEMeD study, to report on the EQ-domains and EQ-VAS scores of advanced elderly (i.e., ≥75 years) in six European countries, including the 164 Dutch responders aged 75 and older. A more recent study (data collected in 2012), reported EQ-5D-5 L index scores for the age-groups 60–70 and ≥70 years based on 281 respondents [11]. Important determinants for health identified in earlier studies were age (e.g. [8, 13–19]), gender (e.g. [8, 13–19]) and education (e.g. [8, 13, 14, 16, 19]). Detailed comorbidity data were in most of these studies lacking.

The aim of this study was to evaluate health status and associated factors in community-dwelling elderly in the Netherlands, using the EQ-5D-3L instrument.

## Methods

### Data collection

Data presented in the current study was collected from the “Cost, Health status and Outcomes of community-acquired pneumonia (CAP)” (CHO-CAP) source population, which consisted of community-dwelling elderly (≥65 years) in the Netherlands. The aim of the CHO-CAP study was to prospectively collect information on health outcomes and costs of CAP cases in a nested matched cohort. Full details on study design are provided elsewhere [20]. The CHO-CAP study was executed in parallel to the “Community-Acquired Pneumonia immunization Trial in Adults” (CAPiTA), a placebo-controlled double-blind randomized clinical trial evaluating the effectiveness of a 13-valent conjugate pneumococcal vaccine in 84,496 community-dwelling elderly in the Netherlands [21, 22]. Overall 72,074 CAPiTA participants were invited at the time of vaccination (November 2008-January 2010) to participate in the CHO-CAP study. The remaining 12,422 subjects (14.7%) participating in CAPiTA were vaccinated previous to the start of the CHO-CAP study. At the time of vaccination, subjects reveived written information on the CHO-CAP study together with a questionnaire. They were asked to provide information on their current health status to complement socio-demographic and comorbidity details collected in CAPiTA, and to return the questionnaire together with a signed informed consent in a pre-stamped envelope. Those who did (*n* = 48,634) formed the CHO-CAP source population and were eligible for participation in a nested matched cohort to prospectively collect information on health outcomes and costs of CAP cases. Key eligibility criteria were: no previous pneumococcal vaccination and absence of protocol-defined immunocompromising conditions (for full details see [22] and Additional file 1: Section 1). In this study we used the data collected from the CHO-CAP source population and the self-reported comorbidities collected within the CAPiTA-trial [22].

### Health status using the EQ-5D-3L instrument

The EQ-5D-3L instrument was developed by the Euroqol group [2]. The instrument consists of two parts, the EQ-5D descriptive system and the EQ-visual analogue scale (VAS). The EQ-VAS records the participant’s self-reported health on a VAS from 0 to 100, with anchor points being labelled as “Best imaginable health state” (100) and “Worst imaginable health state” (0). The EQ-5D-3L descriptive system consists of five domains (i.e. mobility, self-care, usual activities, pain/discomfort and anxiety/depression) and three levels of functioning (i.e. no problems, some problems or severe problems) [2]. The EQ-5D-3L health states were scored with the Dutch value set [23], to obtain EQ-5D-3L summary index values (EQ-5D-index) standardized from 0 (representing death) to 1 (representing full health), with negative values representing states worse than death [2, 23].

### Socio-demographic and comorbidity data

Self-reported socio-demographic data were age, gender, race, living situation and postal code. Self-reported comorbidities included asthma, diabetes with and without use of insulin, heart disease, liver disease, lung disease, history of splenectomy, history of stroke and/or myocardial infarction and smoking behaviour. The postal code was used to determine the geographic region of participants (i.e. east, south, north and west). The Nomenclature of Territorial Units for Statistics (NUTS) was used to determine the regions [24, 25].

### Data analysis

The percentage reporting problems in one or more domains of EQ-5D, the EQ-5D-indices and the EQ-VAS were examined. Differences between age-groups were tested using Chi-square test for categorical outcomes and Mann–Whitney U test or Kruskal Wallis test (≥2 groups) for continuous outcomes. Results were presented by predefined age-groups (65–69; 70–74; 75–79; 80–84 and ≥85) and by sex (male and female).

Furthermore, a stepwise linear regression model was used to identify factors associated with quality-of-life, with EQ-5D-indices and EQ-VAS as dependent variable respectively. Explanatory variables were age (continuous variable), gender, education, region, smoking, having comorbidity *x* ( *x* stands for asthma, diabetes with and without use of insulin, heart disease, liver disease, lung disease and history of splenectomy) and/or having a previous cardiovascular event *y* ( *y* stands for stroke, and myocardial infarction). Analyses were conducted using SPSS version 22.

## Results

The CHO-CAP questionnaire was distributed to 72,074 CAPiTA-participants. The questionnaire and informed consent were received from 48,634 subjects, corresponding to a response rate of 67.5% of those invited to participate (see Additional file 1: Figure S1).

The majority of participants were male (57%), were aged between 65 and 74 years (69.9%), were low educated (41.9%), lived in the west (36.6%) and had no comorbidities (54.8%), see Table 1 and Additional file 1: Table S1. Women, the very elderly, and lower educated elderly were underrepresented in the study population (Table 1). Non-responders had more comorbidities than those included in the study (see Additional file 1: Table S1).

**Table 1:** Characteristics of the study population compared to the elderly (i.e. ≥ 65 years) Dutch population

	CHO-CAP-study (*n* = 48,634)	General Dutch elderly population in 2010^a^ (*n* = 2,538,328)
Sex (%)		
Male	57.2	43.7
Female	42.8	56.3
Age (%)		
65–69 years	39.9	30.6
70–74 years	30.0	24.4
75–79 years	18.3	19.5
80–84 years	8.7	13.8
≥ 85 years	3.1	11.7
Education (%)^b^		
Low	41.9	56.2
Middle	33.8	28.2
High	23.0	15.6
Region (%)		
North	6.2	11.2
East	24.7	20.6
West	36.6	45.1
South	32.4	23.1

^a^ Figures from the Dutch population are derived from Statistics Netherlands for the year 2010 [29]

^b^ Education level: 1.2% missing data

**Fig. 1:**
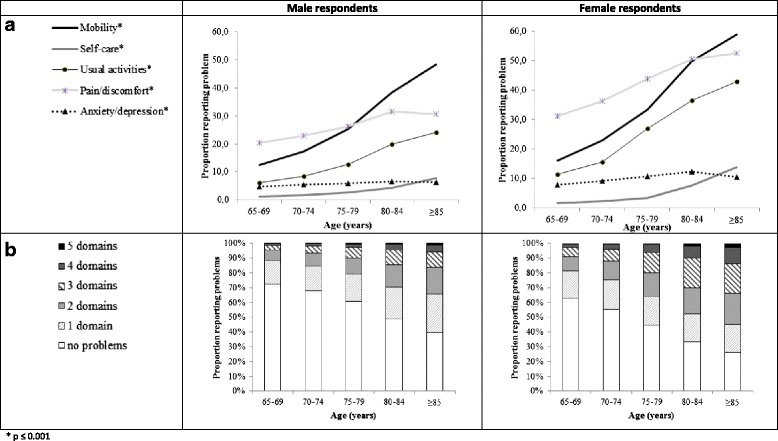
Profile of the population: Percentage reporting any problems per domain (**a**) and percentage reporting number of domains with problems (**b**) by age-group and sex, respectively. * *p* ≤ 0.001

The proportion of elderly with no problems, some problems and severe problems of functioning by domain and by age-group and sex is shown in Fig. 1a with underlying data in Additional file 1: Table S2. The population profile by age-group and sex, expressed as percentage reporting problems in one or several domains is presented in Fig. 1b and in Additional file 1: Figure S2. In most age-classes, pain/discomfort was the most frequently reported complaint, ranging from 24.1% (age-group 65–69) to 38.0% (age-group 80–84). Relatively few respondents had problems with self-care, ranging from 1.4% (age-group 65–69) to 5.6% (age-group 80–84). In the eldest elderly (≥85 years), mobility was the most frequently reported problem (52.9%), while anxiety/depression was least frequently reported as problem (8.1%). The proportion of persons reporting problems, and the number of domains with problems is rising with increasing age (see Figs. 1a–1b). In all age-groups and for all five domains, women reported more problems than men (see Figs. 1a–1b).

**Fig. 2:**
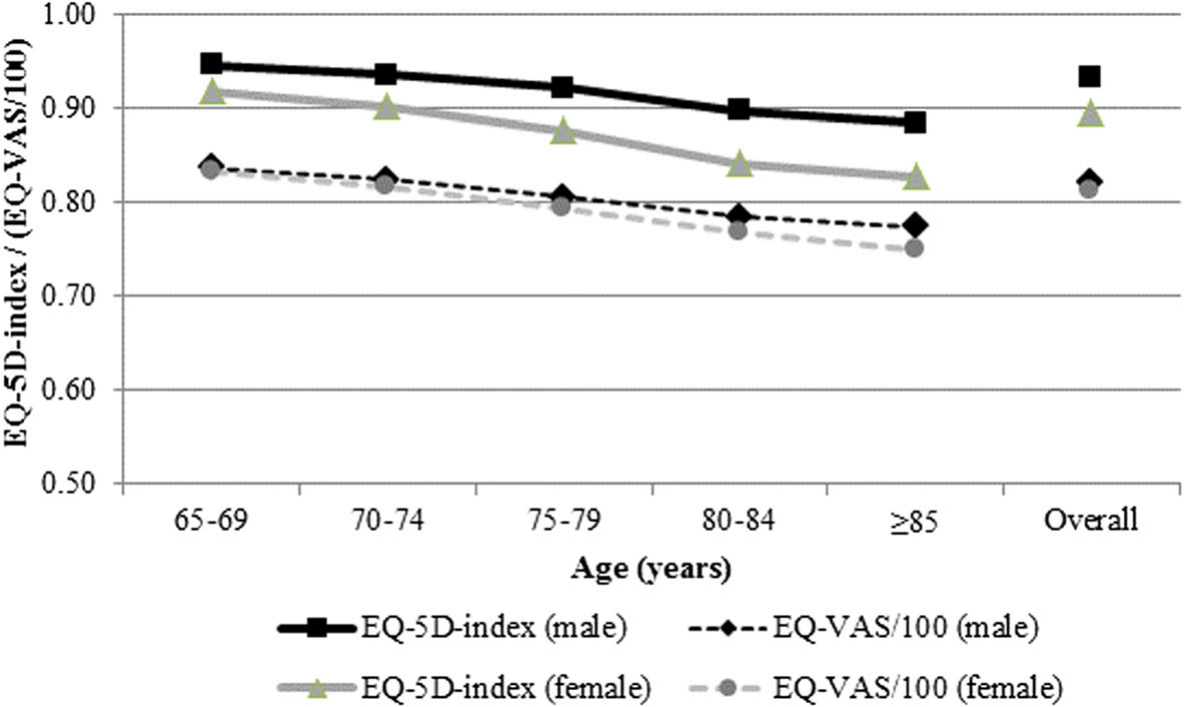
Mean population EQ-VAS and mean EQ-5D-3L-index by age-group and sex. Note: EQ-VAS and EQ-5D-3L indices were significantly different between age-group and sex (*p* > 0.01)

Mean population EQ-VAS and mean EQ-5D-index values were 82 and 0.92, respectively. Male respondents had both, higher EQ-VAS and higher EQ-5D-index values than female respondents, namely 82 versus 81 and 0.93 versus 0.90 respectively, see Fig. 2 and Additional file 1: Tables S3 and S4. Both mean EQ-VAS and EQ-5D-index values decreased significantly with increasing age from 84 to 0.94 in the 65–69 age-group to 76 and 0.86 in the elder elderly, respectively (Fig. 2 and Additional file 1: Tables S3 and S4).

Next to female gender (β = −0.40) and increasing age (β: −0.003), lower education (+0.011 for medium/+0.20 for high education), smoking (−0.021), having a comorbidity (e.g. asthma: − 0.16; diabetes using insulin: −0.027) and/or a previous cardiovascular event (stroke: −0.56; myocardial infarct −0.29) and living in the southern region (−0.005) were negatively associated with EQ-5D index values (see Table 2). Similar findings were found for EQ-VAS. For more details see Table 2.

**Table 2:** Factors associated with health status, with EQ-5D-indices and EQ-VAS as dependent variables, respectively

	Un-standardised regression coefficients (*p*-value)	
	EQ-5D-indices (R^2^ = 0.090)	EQ-VAS (R^2^ = 0.098)
Constant	1.181 (0.000)	106.683 (0.000)
Age (in years)	−0.003 (0.000)	−0.308 (0.000)
Gender (male = reference)	−0.040 (0.000)	−1.672 (0.000)
Education (low = reference)		
Moderate	0.011 (0.000)	0.462 (0.000)
High	0.020 (0.000)	0.575 (0.000)
Region (west = reference)		
North	0.001 (0.606)	0.050 (0.832)
East	−0.002 (0.093)	−0.183 (0.191)
South	−0.005 (0.000)	−0.768 (0.000)
Smoking	−0.021 (0.000)	−1.232 (0.000)
Asthma	−0.016 (0.000)	−1.932 (0.000)
Diabetes – using insulin	−0.027 (0.000)	−3.125 (0.000)
Diabetes – no insulin	−0.010 (0.000)	−1.697 (0.000)
Heart disease	−0.012 (0.000)	−1.633 (0.000)
Lung disease	−0.018 (0.000)	−2.386 (0.000)
Liver disease	- ^a^	0.418 (0.031)
Previous stroke	−0.056 (0.000)	−5.928 (0.000)
Previous myocardial infarct	−0.029 (0.000)	−4.563 (0.000)

^a^ Not significant in univariate analysis therefore not included in multivariate analysis

## Discussion

Within elderly people, large quality-of-life differences exist. The proportion reporting problems was rising with increased age, as was also the proportion of persons reporting problems in more than one domain. For most age-groups and both sexes, pain/discomfort was the most frequently reported complaint. Similar to other studies, self-care problems were reported least frequent [9, 19, 26]. Only in the oldest elderly, mobility problems were more frequently reported than pain/discomfort, while anxiety/depression was the least frequent reported problem. Similar to other studies [8–10, 13–19] female sex was associated with more problems on each of the five EQ-5D domains, and consequently also with a lower EQ-5D-index compared to men. Increasing age, female sex, lower education, smoking, having a comorbidity and/or a previous cardio-vascular event, but also geographic factors such as living in the southern region of the Netherlands and living in an urban area, were all associated with lower EQ-5D health status.

The use of national tariffs, different response styles due to social and cultural background and different reference levels all influence the final EQ-5D-indices score [10, 13, 15, 27]. According to Szende et al. [8] it is mainly the prior living standards of a country explaining the observed cross-country differences in general health. Therefore, a direct comparison with other countries is hampered by these important cross-country differences in background. König et al. [10], who compared the health status of elderly in six European countries, namely Belgium, France, Germany, Italy, the Netherlands, and Spain, noted that the Netherlands was the country with the lowest proportion of respondents reporting any problems. This corresponds to the findings of Konerding et al. [13] who studied the health status in adult type 2 diabetes patients in six European countries, and who noted that the Dutch respondents reported fewer problems in four of the five domains, and only Finland reported fewer problems with depression and/or anxiety. In our study, the tendency of reporting problems, increased with age and was associated with being female and having a low education, similar to other studies (e.g. [6, 8–10, 13–16, 19, 27]). Pain/discomfort was the most frequently reported problem, but in the eldest elderly problems with the mobility domain occurred most frequently, similar to findings in a Dutch study of Hoeymans et al. [9]. Anxiety/depression was reported least frequent like, in other studies [8–10]. Overall, our large sample of respondents appeared to report slightly fewer problems in most domains of EQ-5D compared to previous studies conducted in Dutch elderly [6–8]. The relative largest differences were found for anxiety/depression. According to Szende et al. [8] 9.9% and 12.5% of the 65–74 years and ≥75 years old reported to be moderately or extremely anxious or depressed, and according to Hoeymans et al. [9] this was 11.8% (65–79 years) and 13.6% (≥80 years). In our population only 6% reported to be moderately or extremely anxious or depressed, similar to König et al. [10]. Comparing the mean EQ-5D-index scores found in other Dutch studies, our estimates were slightly higher [6–8] which may be explained from the fact that our sample consisted of a relatively fit and healthy population participating in a clinical trial, with more males and higher education level compared to the general population.

Social and cultural background differences mostly found between countries [10, 13, 15, 27] could be confirmed to exist also within a small country such as the Netherlands. In particular, respondents living in Southern Netherlands tend to have a slightly lower health status than those living in other regions. This is a consistent finding in many studies on within country differences in health status [28]. The different cultural and social background and history of the Southern part of the country seems to have an impact up to today.

Strength of our study is the large study population of elderly persons (i.e. ≥65 years), allowing stratification by gender and five age-groups. A further strength of the current study is the data availability of prevalent chronic comorbidities and/or a previous cardio-vascular event.

One of the limitations of our study was that we had a sample with a higher percentage of men than all other studies and furthermore our population had a higher education level than the general population. By definition, immunocompromised persons were excluded, as this was an exclusion criterion in the CAPiTA-trial [21, 22]. Our study population was therefore probably fitter and healthier than the general Dutch elderly population.

## Conclusion

Within community-dwelling elderly large differences in health status exist. Impairment increases rapidly with age, but health status is also associated with socio-demographic variables and comorbidities.

## Additional file

Additional file 1:
**section 1**- Inclusion and exclusion criteria. **Figure S1** - Flow chart of the CHO-CAP population. **Table S1** - Baseline characteristics of non-responders and CHO-CAP participants. **Table S2** – Proportion of three levels of functioning by domain by age-group as reported by male and female respondents, respectively. **Figure S2** - Profile of the population (all respondents): Percentage reporting problems by age-group. **Table S3** – EQ-VAS scores by age for male, female and total respondents, respectively. **Table S4** –EQ-5D-3L-indices by age for male, female and total respondents, respectively. (DOCX 102 kb)

## Abbreviations

CAP: Community-acquired pneumonia
CAPiTA: Community-acquired pneumonia immunization trial in adults
CHO-CAP: Costs, health status and Outcomes of community-acquired pneumonia
EQ-5D: Euroqol group EQ-5D-3L instrument
EQ-5D-indices: Euroqol group EQ-5D-3L summary index values
EQ-VAS: Euroqol group visual analogue scale
MAUIs: Multi-attribute utility instruments
NUTS: Nomenclature of territorial units for statistics
QALY: Quality-adjusted life years
