# Supplementary material for: Quality of life in community-dwelling Dutch elderly measured by EQ-5D-3L
Source: Health Qual Life Outcomes. 2017 Jan 6;15:3. doi: 10.1186/s12955-016-0577-5 (PMC5220621; doi:10.1186/s12955-016-0577-5)
Supplement: Additional file 1: — section 1- Inclusion and exclusion criteria. Figure S1 - Flow chart of the CHO-CAP population. Table S1 - Baseline characteristics of non-responders and CHO-CAP participants. Table S2 – Proportion of three levels of functioning by domain by age-group as reported by male and female respondents, respectively. Figure S2 - Profile of the population (all respondents): Percentage reporting problems by age-group. Table S3 – EQ-VAS scores by age for male, female and total respondents, respectively. Table S4 –EQ-5D-3L-indices by age for male, female and total respondents, respectively. (DOCX 102 kb) [file 12955_2016_577_MOESM1_ESM.docx]

**Additional file 1**

**Manuscript**

Quality of life in community-dwelling Dutch elderly measured by EQ-5D-3L

**Supplementary Online Content**

**Content of Figures**

[Figure S. 1 - Flow chart of the CHO-CAP population 3](#_Toc432670148)

[Figure S. 2 - Profile of the population (all respondents): Percentage reporting problems by age-group 7](#_Toc432670149)

**Content of Tables**

[Table S. 1 - Baseline characteristics of non-responders and CHO-CAP participants. 4](#_Toc432670153)

[Table S. 2 – Proportion of levels 1, 2 and 3 by domain by age-group as reported by male and female respondents, respectively. 5](#_Toc432670154)

[Table S. 3 – EQ-VAS scores by age for male, female and total respondents, respectively 8](#_Toc432670155)

[Table S. 4 –EQ-5D-3L-indexes by age for male, female and total respondents, respectively 9](#_Toc432670156)

# Inclusion and exclusion criteria

**Inclusion and exclusion criteria of the CAPiTA trial**

Subjects were eligible to participate in the study if they met all of the following inclusion criteria:

1. Male or female adults aged 65 years or older on the date of vaccination.
2. Registered with a GP who was referring subjects to the trial.
3. Able to fulfill study requirements.

Subjects were ineligible to participate in this study if they met any of the following exclusion criteria:

1. Previous vaccination with any licensed or experimental pneumococcal vaccine.
2. Residence in a nursing home, long-term care facility, or other institution, or requirement of semiskilled nursing care. (An ambulatory subject who was a resident of a retirement home or village was eligible for the trial.)
3. Contraindication for vaccination with PCV13.
4. Contraindication for vaccination with influenza vaccine, if influenza vaccine was to be administered.
5. Use of investigational vaccine or medication within 30 days before study vaccine administration.
6. History of severe adverse reaction associated with a vaccine or vaccine component.
7. Immune deficiency or suppression, defined as presence of 1 or more of the following conditions:

• human immunodeficiency virus (HIV) infection

• leukemia (presence defined as having been treated by or been eligible for treatment by radiotherapy and/or chemotherapy within the last 5 years)

• lymphoma (presence defined as having been treated by or been eligible for treatment by radiotherapy and/or chemotherapy within the last 5 years)

• Hodgkin disease (presence defined as having been treated by or been eligible for treatment by radiotherapy and/or chemotherapy within the last 5 years)

• multiple myeloma (presence defined as having been treated by or been eligible for treatment by radiotherapy and/or chemotherapy within the last 5 years)

• generalized malignancy (defined as presence of any malignancy that had been treated by or had been eligible for treatment by radiotherapy and/or chemotherapy within the last 5 years)

• chronic renal failure (defined as receipt of renal dialysis or transplant) or nephrotic syndrome

• receipt of immunosuppressive therapy, including steroids, within 3 months of study vaccine administration (For corticosteroids, this meant prednisone or equivalent, 0.5 mg/kg/day for 14 days. Inhaled, intraarticular, and topical steroids were not considered immunosuppressive.)

• receipt of an organ or bone marrow transplant

**Additional inclusion criteria for the CHO-CAP study**

All CAPiTA participants were eligible for participation in the CHO-CAP study, and were included when:

- answering and returning the distributed CHO-CAP questionnaire, together with a signed informed consent.

Figure S1 - Flow chart of the CHO-CAP population


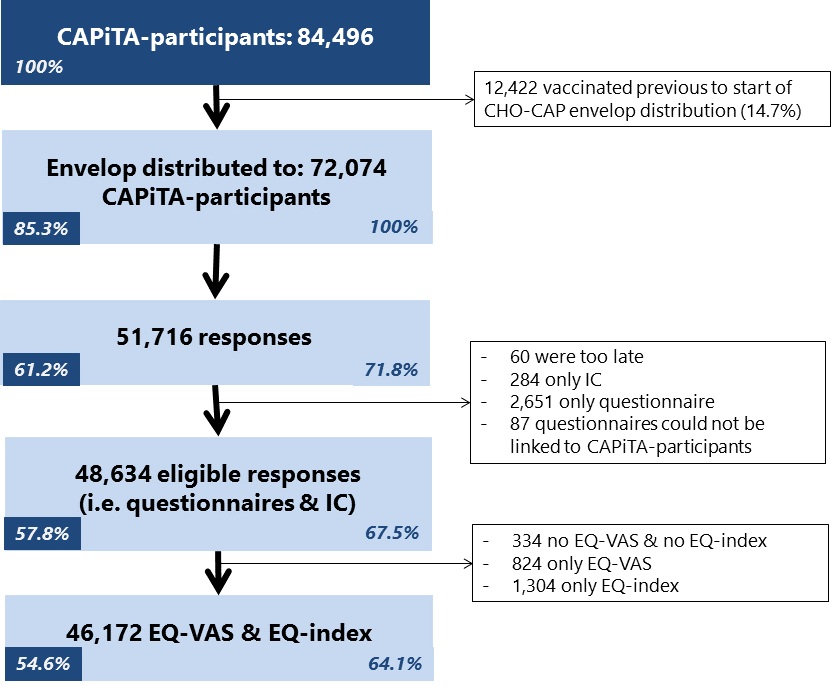


Table S1 - Baseline characteristics of non-responders and CHO-CAP participants.

|  | CHO-CAP-study  *n=48,634* | Non-responders ^#^  *n=35,866* | p-value |
| --- | --- | --- | --- |
| Age in years, median (IQR) | 71 (68-76) | 71 (68-76) | <0.001 |
| Male | 57.16% | 54.24% | <0.001 |
| Race – white | 98.70% | 98.20% | <0.001 |
| Vaccinated with PCV13 | 49.90% | 50.10% | 0.055 |
| Asthma | 4.52% | 5.39% | <0.001 |
| Diabetes mellitus: Insulin use | 2.92% | 3.64% | <0.001 |
| Diabetes mellitus: No insulin | 8.87% | 9.84% | <0.001 |
| Heart disease | 23.56% | 27.80% | <0.001 |
| Liver disease | 0.48% | 0.49% | 0.104 |
| Lung disease | 9.83% | 10.69% | <0.001 |
| Splenectomy | 0.07% | 0.11% | 0.006 |
| Median number of comorbidities (IQR) | 0 (0-1) | 0 (0-1) | <0.001 |
| No comorbidities | 59.81% | 54.80% |  |
| 1 comorbidity | 31.31`% | 33.90% |  |
| 2 comorbidities | 7.78% | 9.90% |  |
| 3 or more comorbidities | 1.09% | 1.40% |  |
| Current smoking | 11.44% | 13.42% | <0.001 |

^#^ Non-responders, including the first14.7% of the “Community-Acquired Pneumonia immunization Trial in Adults” (CAPiTA) participants that could not be approached as the CHO-CAP-study started later than the CAPiTA-study.

Table S2 – Proportion of three levels of functioning by domain by age-group as reported by male and female respondents, respectively.

| EQ-5D domain | | Age, years | | | | | TOTAL |
| --- | --- | --- | --- | --- | --- | --- | --- |
|  |  | **65-69** | **70-74** | **75-79** | **80-84** | **≥85** |  |
| *Male respondents* | | | | | | | |
|  | *n* | *11,155* | *8,085* | *4,885* | *2,333* | *829* | *27,287* |
| Mobility* | No problem | 87.6 | 82.6 | 74.7 | 61.6 | 51.5 | 80.5 |
|  | Some problem | 12.3 | 17.3 | 25.3 | 38.3 | 48.4 | 19.4 |
|  | Severe problems | 0.1 | 0.1 | 0.0 | 0.1 | 0.1 | 0.1 |
| Self-care* | No problem | 98.8 | 98.4 | 97.3 | 95.8 | 92.3 | 97.9 |
|  | Some problem | 1.1 | 1.4 | 2.6 | 3.9 | 7.4 | 1.9 |
|  | Severe problems | 0.1 | 0.2 | 0.1 | 0.3 | 0.4 | 0.1 |
| Usual activities* | No problem | 93.9 | 91.4 | 87.3 | 80.2 | 75.9 | 90.2 |
|  | Some problem | 5.9 | 8.4 | 12.1 | 18.7 | 23.4 | 9.4 |
|  | Severe problems | 0.2 | 0.2 | 0.6 | 1.1 | 0.7 | 0.4 |
| Pain / discomfort* | No problem | 79.6 | 77.0 | 73.7 | 68.5 | 69.4 | 76.5 |
|  | Some problem | 19.9 | 22.6 | 25.9 | 30.7 | 29.9 | 23.0 |
|  | Severe problems | 0.5 | 0.5 | 0.5 | 0.8 | 0.7 | 0.5 |
| Anxiety / Depression* | No problem | 95.3 | 94.6 | 94.1 | 93.5 | 93.6 | 94.7 |
|  | Some problem | 4.6 | 5.3 | 5.8 | 6.5 | 6.4 | 5.2 |
|  | Severe problems | 0.1 | 0.1 | 0.1 | 0.0 | 0 | 0.1 |
| *Female respondents* | | | | | | | |
|  | *n* | *7,996* | *6,181* | *3,691* | *1,715* | *606* | *20,189* |
| Mobility* | No problem | 84.0 | 77.1 | 66.7 | 50.1 | 41.1 | 74.5 |
|  | Some problem | 16.0 | 22.8 | 33.3 | 49.7 | 58.9 | 25.4 |
|  | Severe problems | 0.1 | 0.1 | 0.1 | 0.2 | 0 | 0.1 |
| Self-care* | No problem | 98.4 | 97.6 | 96.6 | 92.5 | 86.1 | 97.0 |
|  | Some problem | 1.4 | 2.2 | 3.1 | 6.9 | 13.4 | 2.8 |
|  | Severe problems | 0.2 | 0.2 | 0.3 | 0.6 | 0.5 | 0.2 |
| Usual activities* | No problem | 88.5 | 84.5 | 73.1 | 63.6 | 57.1 | 81.4 |
|  | Some problem | 11.1 | 15.0 | 26.1 | 34.9 | 41.3 | 18.0 |
|  | Severe problems | 0.3 | 0.5 | 0.8 | 1.6 | 1.7 | 0.6 |
| Pain / discomfort* | No problem | 68.9 | 63.6 | 56.1 | 49.6 | 47.5 | 62.7 |
|  | Some problem | 30.0 | 34.9 | 42.6 | 47.9 | 50.5 | 35.9 |
|  | Severe problems | 1.1 | 1.4 | 1.3 | 2.4 | 2.0 | 1.4 |
| Anxiety / Depression* | No problem | 92.1 | 90.8 | 89.2 | 87.8 | 89.6 | 90.7 |
|  | Some problem | 7.7 | 9.0 | 10.5 | 12.0 | 10.4 | 9.1 |
|  | Severe problems | 0.2 | 0.2 | 0.3 | 0.2 | 0 | 0.2 |
|  | | | | | | | |
| *All respondents* | | | | | | | |
|  | *n* | *19,151* | *14,266* | *8,576* | *4,048* | *1,435* | *47,476* |
| Mobility* | No problem | 86.1 | 80.2 | 71.2 | 56.7 | 47.1 | 78.0 |
|  | Some problem | 13.8 | 19.7 | 28.7 | 43.1 | 52.8 | 22.0 |
|  | Severe problems | 0.1 | 0.1 | 0.0 | 0.1 | 0.1 | 0.1 |
| Self-care* | No problem | 98.6 | 98.1 | 97.0 | 94.4 | 89.7 | 97.5 |
|  | Some problem | 1.3 | 1.8 | 2.8 | 5.2 | 9.9 | 2.3 |
|  | Severe problems | 0.1 | 0.2 | 0.2 | 0.4 | 0.4 | 0.2 |
| Usual activities* | No problem | 91.7 | 88.4 | 81.2 | 73.1 | 67.9 | 86.5 |
|  | Some problem | 8.1 | 11.3 | 18.1 | 25.6 | 30.9 | 13.0 |
|  | Severe problems | 0.3 | 0.3 | 0.7 | 1.3 | 1.1 | 0.5 |
| Pain / discomfort* | No problem | 75.7 | 71.2 | 66.1 | 60.5 | 60.1 | 70.6 |
|  | Some problem | 24.1 | 27.9 | 33.1 | 38.0 | 38.6 | 28.5 |
|  | Severe problems | 0.8 | 0.9 | 0.8 | 1.5 | 1.3 | 0.9 |
| Anxiety / Depression* | No problem | 94.0 | 92.9 | 92.0 | 91.1 | 91.9 | 93.8 |
|  | Some problem | 5.9 | 6.9 | 7.8 | 8.8 | 8.1 | 6.8 |
|  | Severe problems | 0.1 | 0.2 | 0.2 | 0.1 | 0 | 0.1 |

* p<0.001.

Figure S2 - Profile of the population (all respondents): Percentage reporting problems by age-group


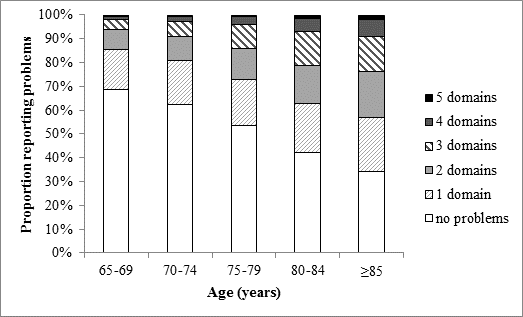


Note: profile of the population was significant different (p<0.001) between age-groups.

Table S3 – EQ-VAS scores by age for male, female and total respondents, respectively

|  | Age (in years) | | | | | Total |
| --- | --- | --- | --- | --- | --- | --- |
| EQ-VAS | 65-69 | 70-74 | 75-79 | 80-84 | ≥85 |  |
| *Male respondents* | | | | | | |
| *n* | *10,989* | *7,978* | *4,846* | *2,317* | *818* | *26,948* |
| Mean | 83.69 | 82.44 | 80.55 | 78.42 | 77.36 | 82.11 |
| StD | 11.25 | 11.60 | 11.90 | 12.39 | 13.02 | 11.77 |
| Median | 85.00 | 80.00 | 80.00 | 80.00 | 80.00 | 80.00 |
| 25% | 77.00 | 75.00 | 75.00 | 70.00 | 70.00 | 75.00 |
| 75% | 90.00 | 90.00 | 90.00 | 89.00 | 88.25 | 90.00 |
| *Female respondents* | | | | | | |
| *n* | *7,879* | *6,147* | *3,684* | *1,734* | *604* | *20,048* |
| Mean | 83.23 | 81.64 | 79.13 | 76.56 | 74.99 | 81.16 |
| StD | 11.82 | 12.25 | 12.97 | 13.03 | 14.24 | 12.57 |
| Median | 80.00 | 80.00 | 80.00 | 78.00 | 75.00 | 80.00 |
| 25% | 75.00 | 75.00 | 70.00 | 70.00 | 69.00 | 75.00 |
| 75% | 90.00 | 90.00 | 90.00 | 85.00 | 85.00 | 90.00 |
| *All respondents* | | | | | | |
| *n* | *18,868* | *14,125* | *8,530* | *4,051* | *1,422* | *46,996* |
| Mean | 83.50 | 82.10 | 79.94 | 77.62 | 76.35 | 81.71 |
| StD | 11.50 | 11.89 | 12.39 | 12.70 | 13.60 | 12.13 |
| Median | 85.00 | 80.00 | 80.00 | 80.00 | 78.00 | 80.00 |
| 25% | 76.00 | 75.00 | 70.00 | 70.00 | 70.00 | 75.00 |
| 75% | 90.00 | 90.00 | 90.00 | 85.00 | 85.00 | 90.00 |

Note: EQ-VAS scores were significantly different between age-groups (p>0.001); between sex (p>0.01) and between age-group and sex (p>0.01).

Table S4 –EQ-5D-3L-indices by age for male, female and total respondents, respectively

|  | Age (in years) | | | | | Total |
| --- | --- | --- | --- | --- | --- | --- |
| EQ-5D-index | 65-69 | 70-74 | 75-79 | 80-84 | ≥85 |  |
| *Male respondents* | | | | | | |
| *n* | *11,155* | *8,085* | *4,885* | *2,333* | *829* | *27,287* |
| Mean | 0.95 | 0.94 | 0.92 | 0.90 | 0.89 | 0.93 |
| StD | 0.11 | 0.11 | 0.12 | 0.13 | 0.12 | 0.11 |
| Median | 1.00 | 1.00 | 1.00 | 0.90 | 0.89 | 1.00 |
| 25% | 0.89 | 0.84 | 0.84 | 0.81 | 0.81 | 0.84 |
| 75% | 1.00 | 1.00 | 1.00 | 1.00 | 1.00 | 1.00 |
| *Female respondents* | | | | | | |
| *n* | *7,996* | *6,181* | *3,691* | *1,715* | *606* | *20,189* |
| Mean | 0.92 | 0.90 | 0.88 | 0.84 | 0.83 | 0.90 |
| StD | 0.13 | 0.14 | 0.14 | 0.16 | 0.15 | 0.14 |
| Median | 1.00 | 1.00 | 0.89 | 0.84 | 0.81 | 1.00 |
| 25% | 0.84 | 0.81 | 0.81 | 0.78 | 0.78 | 0.81 |
| 75% | 1.00 | 1.00 | 1.00 | 1.00 | 1.00 | 1.00 |
| *All respondents* | | | | | | |
| *n* | *19,151* | *14,266* | *8,576* | *4,048* | *1,435* | *47,476* |
| Mean | 0.93 | 0.92 | 0.90 | 0.87 | 0.86 | 0.92 |
| StD | 0.12 | 0.12 | 0.13 | 0.15 | 0.14 | 0.13 |
| Median | 1.00 | 1.00 | 1.00 | 0.89 | 0.89 | 1.00 |
| 25% | 0.84 | 0.84 | 0.81 | 0.81 | 0.78 | 0.84 |
| 75% | 1.00 | 1.00 | 1.00 | 1.00 | 1.00 | 1.00 |

Note: EQ-5D-3L indices were significantly different between age-groups (p>0.001); between sex (p>0.01) and between age-group and sex (p>0.01).
